# Trauma and PTSD in the WHO World Mental Health Surveys

**DOI:** 10.1080/20008198.2017.1353383

**Published:** 2017-10-27

**Authors:** Ronald C. Kessler, Sergio Aguilar-Gaxiola, Jordi Alonso, Corina Benjet, Evelyn J. Bromet, Graça Cardoso, Louisa Degenhardt, Giovanni de Girolamo, Rumyana V. Dinolova, Finola Ferry, Silvia Florescu, Oye Gureje, Josep Maria Haro, Yueqin Huang, Elie G. Karam, Norito Kawakami, Sing Lee, Jean-Pierre Lepine, Daphna Levinson, Fernando Navarro-Mateu, Beth-Ellen Pennell, Marina Piazza, José Posada-Villa, Kate M. Scott, Dan J. Stein, Margreet Ten Have, Yolanda Torres, Maria Carmen Viana, Maria V. Petukhova, Nancy A. Sampson, Alan M. Zaslavsky, Karestan C. Koenen

**Affiliations:** ^a^ Department of Health Care Policy, Harvard Medical School, Boston, MA, USA; ^b^ Center for Reducing Health Disparities, UC Davis Health System, Sacramento, CA, USA; ^c^ Health Services Research Unit, IMIM-Hospital del Mar Medical Research Institute, Barcelona, Spain; ^d^ Departament de Ciències Experimentals i de la Salut, Pompeu Fabra University, Barcelona, Spain; ^e^ CIBER en Epidemiología y Salud Pública (CIBERESP), Barcelona, Spain; ^f^ Department of Epidemiologic and Psychosocial Research, National Institute of Psychiatry Ramón de la Fuente Muniz, Mexico City, Mexico; ^g^ Department of Psychiatry, Stony Brook University School of Medicine, Stony Brook, NY, USA; ^h^ Lisbon Institute of Global Mental Health and Chronic Diseases Research Center (CEDOC), Nova Medical School, Universidade Nova de Lisboa, Lisbon, Portugal; ^i^ National Drug and Alcohol Research Centre, University of New South Wales, Sydney, Australia; ^j^ Unit of Epidemiological and Evaluation Psychiatry, Istituti di Ricovero e Cura a Carattere Scientifico (IRCCS)-St. John of God Clinical Research Centre, Brescia, Italy; ^k^ Sector “Mental Health”, National Center of Public Health and Analyses, Sofia, Bulgaria; ^l^ Bamford Centre for Mental Health and Wellbeing, Ulster University, Northern Ireland; ^m^ National School of Public Health, Management and Development, Bucharest, Romania; ^n^ Department of Psychiatry, University College Hospital, Ibadan, Nigeria; ^o^ Parc Sanitari Sant Joan de Déu, CIBERSAM, Universitat de Barcelona, Sant Boi de Llobregat, Barcelona, Spain; ^p^ Institute of Mental Health, Peking University, Beijing, People’s Republic of China; ^q^ Department of Psychiatry and Clinical Psychology, Faculty of Medicine, Balamand University, Beirut, Lebanon; ^r^ Department of Psychiatry and Clinical Psychology, St George Hospital University Medical Center, Beirut, Lebanon; ^s^ Institute for Development, Research, Advocacy and Applied Care (IDRAAC), Beirut, Lebanon; ^t^ Department of Mental Health, School of Public Health, The University of Tokyo, Tokyo, Japan; ^u^ Department of Psychiatry, Chinese University of Hong Kong, Tai Po, Hong Kong; ^v^ Psychiatrie non sectorisée, Hôpital Lariboisière- Fernand Widal, Assistance Publique Hôpitaux de Paris, Paris, France; ^w^ INSERM UMR-S 1144, Universités Paris Descartes-Paris Diderot, Paris, France; ^x^ Mental Health Services, Ministry of Health, Jerusalem, Israel; ^y^ UDIF-SM, Subdirección General de Planificación, Innovación y Cronicidad, Servicio Murciano de Salud, IMIB-Arrixaca, CIBERESP-Murcia, Murcia, Spain; ^z^ Survey Research Center, Institute for Social Research, University of Michigan, Ann Arbor, MI, USA; ^aa^ Departamento Académico de Salud Pública, Administración y Ciencias Sociales, Universidad Peruana Cayetano Heredia, Lima, Peru; ^ab^ La Unidad de Análisis y Generación de Evidencias en Salud Pública - UNAGESP, National Institute of Health, Lima, Peru; ^ac^ Colegio Mayor de Cundinamarca University, Faculty of Social Sciences, Bogotá, Colombia; ^ad^ Department of Psychological Medicine, University of Otago, Dunedin, New Zealand; ^ae^ Department of Psychiatry and Mental Health, University of Cape Town, Cape Town, Republic of South Africa; ^af^ Department of Epidemiology, Trimbos-Instituut, Netherlands Institute of Mental Health and Addiction, Utrecht, Netherlands; ^ag^ Center for Excellence on Research in Mental Health, CES University, Medellin, Colombia; ^ah^ Department of Social Medicine, Federal University of Espírito Santo, Vitoria, Brazil; ^ai^ Department of Epidemiology, Harvard T.H. Chan School of Public Health, Boston, MA, USA

**Keywords:** Burden of illness, disorder prevalence and persistence, epidemiology, post-traumatic stress disorder (PTSD), trauma exposure

## Abstract

**Background**: Although post-traumatic stress disorder (PTSD) onset-persistence is thought to vary significantly by trauma type, most epidemiological surveys are incapable of assessing this because they evaluate lifetime PTSD only for traumas nominated by respondents as their ‘worst.’

**Objective**: To review research on associations of trauma type with PTSD in the WHO World Mental Health (WMH) surveys, a series of epidemiological surveys that obtained representative data on trauma-specific PTSD.

**Method**: WMH Surveys in 24 countries (n = 68,894) assessed 29 lifetime traumas and evaluated PTSD twice for each respondent: once for the ‘worst’ lifetime trauma and separately for a randomly-selected trauma with weighting to adjust for individual differences in trauma exposures. PTSD onset-persistence was evaluated with the WHO Composite International Diagnostic Interview.

**Results**: In total, 70.4% of respondents experienced lifetime traumas, with exposure averaging 3.2 traumas per capita. Substantial between-trauma differences were found in PTSD onset but less in persistence. Traumas involving interpersonal violence had highest risk. Burden of PTSD, determined by multiplying trauma prevalence by trauma-specific PTSD risk and persistence, was 77.7 person-years/100 respondents. The trauma types with highest proportions of this burden were rape (13.1%), other sexual assault (15.1%), being stalked (9.8%), and unexpected death of a loved one (11.6%). The first three of these four represent relatively uncommon traumas with high PTSD risk and the last a very common trauma with low PTSD risk. The broad category of intimate partner sexual violence accounted for nearly 42.7% of all person-years with PTSD. Prior trauma history predicted both future trauma exposure and future PTSD risk.

**Conclusions**: Trauma exposure is common throughout the world, unequally distributed, and differential across trauma types with respect to PTSD risk. Although a substantial minority of PTSD cases remits within months after onset, mean symptom duration is considerably longer than previously recognized.

## 1. Introduction

The fact that only a small minority of people in the population develops post-traumatic stress disorder (PTSD) (Atwoli, Stein, Koenen, & McLaughlin, 2015) even though the vast majority are exposed to traumas at some time in their life (Benjet et al., 2016) has raised questions about individual differences in psychological vulnerability to PTSD. These questions are the subject of considerable research (Liberzon & Abelson, 2016; Sayed, Iacoviello, & Charney, 2015; Smoller, 2016). One prior consideration is the possibility that PTSD risk varies significantly by trauma type. Such differences have been documented, with highest PTSD risk thought to occur after traumas involving interpersonal violence (Caramanica, Brackbill, Stellman, & Farfel, 2015; Fossion et al., 2015). A related line of research suggests that trauma history is a risk factor for subsequent PTSD, with prior traumas involving violence again possibly of special importance (Lowe, Walsh, Uddin, Galea, & Koenen, 2014; Smith, Summers, Dillon, & Cougle, 2016). Few studies estimating these differences across trauma types did so using unbiased methods, raising questions about the validity of results regarding these differences. The issue of biasedness comes up because of a common data collection convention in general population epidemiological studies of PTSD whereby respondents are asked about lifetime exposure to each of a wide range of traumas but then assessed for PTSD only for the one trauma nominated by the respondent as their worst or most upsetting lifetime trauma. This approach makes it impossible to estimate conditional risk of PTSD after trauma exposure without upward bias because the traumas for which PTSD is assessed are atypically severe.

One approach to deal with this problem is to assess PTSD twice for epidemiological survey respondents who report experiencing multiple lifetime traumas: once for the trauma nominated by the respondent as their *worst* lifetime trauma and a second time for a *random* trauma (i.e. one randomly-selected occurrence of one randomly-selected trauma type). By weighting the random trauma reports by the inverse of their probabilities of selection at the individual level and combining these weighted reports with reports about the worst lifetime trauma, a representative sample can be generated of all lifetime traumas experienced by all survey respondents. The weight would be 1 for respondents who reported lifetime exposure to only one occurrence of one trauma type.

This weighted sample of trauma occurrences can then be used to obtain estimates both of the distribution of trauma exposure in the population and the conditional probability of PTSD after exposure to traumas of different types. These estimates are unbiased if the model is correct, although they will still be subject to the biases of recall error. The current paper reports data on between-trauma differences in the distribution of trauma exposure by trauma type and the risk of PTSD associated with each trauma type in the WHO World Mental Health (WMH) surveys. The WMH Surveys are a series of community epidemiological surveys that used this weighting scheme to generate a representative sample of trauma occurrences in the general population of participating countries (Liu et al., 2017).

We begin by reporting results on overall lifetime prevalence and basic socio-demographic correlates of trauma exposure in the population. We break down traumas into a number of different types for this purpose. We then examine conditional risk of PTSD after trauma exposure and compare this risk across trauma types. We also examine a number of predictors of PTSD risk among people exposed to traumas controlling for trauma type, including socio-demographic predictors, information about prior trauma exposure, and information about history of mental disorder before exposure to the focal trauma. Next, we examine data on the typical course of PTSD; that is, on the duration of PTSD episodes. We examine the same range of predictors of duration as for onset. Finally, we combine all of the above information into a consolidated portrait of the population burden of PTSD broken down by trauma type. This consolidated portrait takes into consideration differences across traumas in prevalence, conditional risk of PTSD, and the duration of PTSD.

## 2. Methods and materials

### 2.1. Sample

The WMH Surveys are a coordinated series of cross-national community epidemiological surveys using consistent sampling, field procedures, and instruments designed to facilitate pooled cross-national analyses of prevalence and correlates of common mental disorders (Kessler & Ustun, 2008). The subset of 26 WMH Surveys considered in this paper are those that assessed lifetime PTSD after both worst and *random traumas*. Five of the surveys were carried out in low/lower-middle income countries (People’s Republic of China [PRC], Colombia, Nigeria, Peru, Ukraine), seven in upper-middle income countries (Brazil, Bulgaria, Colombia [administered after the previously-mentioned Colombian survey, when the country income rating had increased], Lebanon, Mexico, Romania, South Africa), and 14 in high income countries (Australia, Belgium, France, Germany, Israel, Italy, Japan, Netherlands, New Zealand, Northern Ireland, Portugal, Spain [separate national and regional surveys], USA). Each survey was based on a multi-stage clustered area probability sample of adult household residents. Three of the 26 surveys included the urbanized area of their countries (Colombia, Mexico, Peru), and five were based in specific Metropolitan areas (Beijing and Shanghai, PRC; Sao Paulo, Brazil; Medellin, Colombia; Murcia, Spain; six cities in Japan). The Nigerian survey was restricted to specific regions and the other 17 the entire country. More details about the samples are presented in Table 1 (Stein, de Jonge, Kessler, & Scott, in press).

**Table 1.** WMH sample characteristics by World Bank income categories.^a^

					Sample size		
Country by income category	Survey^b^	Sample characteristics^c^	Field dates	Age range	Part I	Part II	Response rate^d^
**I. Low and lower-middle income countries**							
Colombia	NSMH	All urban areas of the country (approximately 73% of the total national population).	2003	18–65	4426	2381	87.7
Nigeria	NSMHW	21 of the 36 states in the country, representing 57% of the national population. The surveys were conducted in Yoruba, Igbo, Hausa, and Efik languages.	2002–2004	18–100	6752	2143	79.3
PRC^e^ – Beijing/Shanghai	B-WMH/S-WMH	Beijing and Shanghai metropolitan areas.	2001–2003	18–70	5201	1628	74.7
Peru	EMSMP	Five urban areas of the country (approximately 38% of the total national population).	2004–2005	18–65	3930	1801	90.2
Ukraine	CMDPSD	Nationally representative.	2002	18–91	4724	1719	78.3
**TOTAL**					(25,033)	(9672)	81.0
**II. Upper-middle income countries**							
Brazil – São Paulo	São Paulo Megacity	São Paulo metropolitan area.	2005–2008	18–93	5037	2942	81.3
Bulgaria	NSHS	Nationally representative.	2002–2006	18–98	5318	2233	72.0
Colombia – Medellin^g^	MMHHS	Medellin metropolitan area.	2011–2012	19–65	3261	1673	97.2
Lebanon	LEBANON	Nationally representative.	2002–2003	18–94	2857	1031	70.0
Mexico	M-NCS	All urban areas of the country (approximately 75% of the total national population).	2001–2002	18–65	5782	2362	76.6
Romania	RMHS	Nationally representative.	2005–2006	18–96	2357	2357	70.9
South Africa^f^	SASH	Nationally representative.	2002–2004	18–92	4315	4315	87.1
**TOTAL**					(28,927)	(16,913)	78.5
**III. High-income countries**							
Australia^f^	NSMHWB	Nationally representative.	2007	18–85	8463	8463	60.0
Belgium	ESEMeD	Nationally representative. The sample was selected from a national register of Belgium residents.	2001–2002	18–95	2419	1043	50.6
France	ESEMeD	Nationally representative. The sample was selected from a national list of households with listed telephone numbers.	2001–2002	18–97	2894	1436	45.9
Germany	ESEMeD	Nationally representative.	2002–2003	19–95	3555	1323	57.8
Israel	NHS	Nationally representative.	2003–2004	21–98	4859	4859	72.6
Italy	ESEMeD	Nationally representative. The sample was selected from municipality resident registries.	2001–2002	18–100	4712	1779	71.3
Japan	WMHJ	Eleven metropolitan areas.	2002–2006	20–98	4129	1682	55.1
Netherlands	ESEMeD	Nationally representative. The sample was selected from municipal postal registries.	2002–2003	18–95	2372	1094	56.4
New Zealand^f^	NZMHS	Nationally representative.	2004–2005	18–98	12,790	7312	73.3
Northern Ireland	NISHS	Nationally representative.	2005–2008	18–97	4340	1986	68.4
Portugal	NMHS	Nationally representative.	2008–2009	18–81	3849	2060	57.3
Spain	ESEMeD	Nationally representative.	2001–2002	18–98	5473	2121	78.6
Spain – Murcia	PEGASUS- Murcia	Murcia region. Regionally representative.	2010–2012	18–96	2621	1459	67.4
USA	NCS-R	Nationally representative.	2001–2003	18–99	9282	5692	70.9
**TOTAL**					(71,758)	(42,309)	64.8
**IV. TOTAL**					(125,718)	(68,894)	70.4

^a^World Bank (2012) Data. Retrieved from http://data.worldbank.org/country. Some of the WMH countries have moved into new income categories since the surveys were conducted. The income groupings above reflect the status of each country at the time of data collection. The current income category of each country is available at the preceding URL.

^b^NSMH (The Colombian National Study of Mental Health); NSMHW (The Nigerian Survey of Mental Health and Wellbeing); B-WMH (The Beijing World Mental Health Survey); S-WMH (The Shanghai World Mental Health Survey); EMSMP (La Encuesta Mundial de Salud Mental en el Peru); CMDPSD (Comorbid Mental Disorders during Periods of Social Disruption); NSHS (Bulgaria National Survey of Health and Stress); MMHHS (Medellín Mental Health Household Study); LEBANON (Lebanese Evaluation of the Burden of Ailments and Needs of the Nation); M-NCS (The Mexico National Comorbidity Survey); RMHS (Romania Mental Health Survey); SASH (South Africa Health Survey); NSMHWB (National Survey of Mental Health and Wellbeing); ESEMeD (The European Study Of The Epidemiology Of Mental Disorders); NHS (Israel National Health Survey); WMHJ 2002–2006 (World Mental Health Japan Survey); NZMHS (New Zealand Mental Health Survey); NISHS (Northern Ireland Study of Health and Stress); NMHS (Portugal National Mental Health Survey); PEGASUS-Murcia (Psychiatric Enquiry to General Population in Southeast Spain-Murcia);NCS-R (The US National Comorbidity Survey Replication).

^c^Most WMH Surveys are based on stratified multistage clustered area probability household samples in which samples of areas equivalent to counties or municipalities in the US were selected in the first stage followed by one or more subsequent stages of geographic sampling (e.g. towns within counties, blocks within towns, households within blocks) to arrive at a sample of households, in each of which a listing of household members was created and one or two people were selected from this listing to be interviewed. No substitution was allowed when the originally sampled household resident could not be interviewed. These household samples were selected from Census area data in all countries other than France (where telephone directories were used to select households) and the Netherlands (where postal registries were used to select households). Several WMH Surveys (Belgium, Germany, Italy, Spain-Murcia) used municipal or universal health-care registries to select respondents without listing households. The Japanese sample is the only totally un-clustered sample, with households randomly-selected in each of the 11 metropolitan areas and one random respondent selected in each sample household: 17 of the 27 surveys are based on nationally representative household samples.

^d^The response rate is calculated as the ratio of the number of households in which an interview was completed to the number of households originally sampled, excluding from the denominator households known not to be eligible either because of being vacant at the time of initial contact or because the residents were unable to speak the designated languages of the survey. The weighted average response rate is 70.4%

^e^People’s Republic of China.

^f^For the purposes of cross-national comparisons we limit the sample to those 18+.

^g^Colombia moved from the ‘lower and lower-middle income’ to the ‘upper-middle income’ category between 2003 (when the Colombian National Study of Mental Health was conducted) and 2010 (when the Medellin Mental Health Household Study was conducted), hence Colombia’s appearance in both income categories. For more information, please see footnote ^a^.

WMH interviews were administered face-to-face in respondent homes by trained lay interviewers after obtaining informed consent using procedures approved by local Institutional Review Boards. The response rate had a weighted (by sample size) mean of 70.4% across surveys (between 45.9% in France and 97.2% in Medellin). The interviews were in two parts. Part I, administered to all respondents (*n* = 125,718), assessed core DSM-IV mental disorders. Part II, administered to all Part I respondents with core disorders and a probability subsample of other respondents (*n* = 68,894), assessed additional disorders and correlates. Traumas and PTSD were assessed in Part II. The analysis sample considered here includes the 50,855 Part II respondents who reported lifetime trauma exposure. As detailed elsewhere (Heeringa et al., 2008), this sample was weighted to match population geographic/socio-demographic distributions and to adjust for under-sampling of Part I non-cases.

### 2.2. Measures

**Traumas**: A total of 29 trauma types were assessed, with reports of lifetime exposure followed by questions about number of lifetime occurrences and age at first occurrence of each type. Traumas were divided into seven categories for purposes of analysis: seven related to war (e.g. combatant, civilian in war zone, relief worker, refugee); four related to physical violence (e.g. physically abused by a caregiver as a child, mugged); four related to intimate partner or sexual violence (raped, sexually assaulted, stalked, physically abused by a romantic partner); seven related to accidents (toxic chemical spill, other man-made disaster, natural disaster, life-threatening motor vehicle collision, other accident where the respondent accidentally caused serious injury to another person, other life-threatening accident, life-threatening illness); unexpected or traumatic death of a loved one; four related to traumas that happened to other people (child had life-threatening illness, other traumas that occurred to loved ones, witnessed physical fights at home as a child, witnessed any other trauma); and a residual category of ‘other’ traumas. The latter category included responses to two questions: (i) an open-ended question: *Did you ever experience any other extremely traumatic or life-threatening event that I haven’t asked you about?* that was backcoded into the other six categories whenever possible and the residual reports coded in an ‘other’ category; and (ii) a question about a ‘private’ trauma: *Sometimes people have experiences they don’t want to talk about in interviews. I won’t ask you to describe anything like this but, without telling me what it was, did you ever have a traumatic event that you didn’t tell me about because you didn’t want to talk about it?* As shown below, a surprisingly large number of respondents answered this question affirmatively.

**PTSD**: DSM-IV PTSD was assessed with the Composite International Diagnostic Interview (CIDI) (Kessler & Ustün, 2004), a fully-structured lay interview that assesses a wide range of common mental disorders. As noted in the Introduction, PTSD was assessed separately for one random occurrence of one randomly-selected trauma type reported by each respondent selected using a random numbers table for that respondent (the respondent’s *random trauma*) and separately for the respondent’s self-reported *worst* trauma. When DSM-IV/CIDI criteria for PTSD were met, the respondent was asked how long symptoms persisted and if the symptoms were still present at the time of interview. Clinical reappraisal interviews with the SCID (Haro et al., 2006) blinded to CIDI diagnoses of PTSD (but instructed to focus on the same trauma as the one assessed in the CIDI in order to guarantee valid comparison of diagnoses) documented moderate CIDI–SCID concordance (Landis & Koch, 1977) (AUC = .69). Sensitivity and specificity were 38.3% and 99.1%, respectively, resulting in a likelihood ratio positive (Sensitivity/[1-Specificity]) of 42.0 that is well above the 10.0 typically considered definitive for a positive screen (Gardner & Altman, 2000). Based on these operating characteristics, a very high proportion of CIDI cases (86.1%) were confirmed by the SCID.

### 2.3. Analysis methods

Cross-tabulations were used to estimate the distribution of lifetime trauma exposure at the individual level and to examine the distribution of trauma exposure as well as conditional risk of PTSD associated with each trauma type in this trauma-level dataset. Means were calculated within subsamples to estimate average number of trauma occurrences given any. Discrete-time survival analysis with time-varying predictors was used to examine the socio-demographic and prior trauma predictors of each type of trauma exposure and the predictors of persistence of PTSD among cases. As noted in the Introduction, random trauma reports were weighted by the inverse of random trauma probability of selection multiplied by the respondent’s Part II weight to generate a sample representative of all traumas experienced by all respondents. Logistic regression analysis was used to examine predictors of conditional risk of PTSD among the trauma-exposed. Design-adjusted standard errors were used to assess significance of individual predictors and design-based Wald χ^2^ tests to evaluate the significance of predictor sets.

## 3. Results

### 3.1. Prevalence and distribution of trauma exposure

Lifetime exposure to one or more traumas was reported by a weighted 70.4% of Part II WMH respondents (*n* = 50,855, with 51,196 random and/or worst events; Table 2). Mean number of lifetime trauma types among those with any was 2.9, for 2.0 trauma types for capita (i.e. .704 × 2.9). The distribution of number of types among respondents with any was 32.1% one, 23.4% two, 16.6% three, 10.7% four, 6.5% five, 3.9% six, 2.5% seven, and 4.3% more than seven. By far the most common trauma types were unexpected death of a loved one (reported by 31.4% of respondents) and direct exposure to (i.e. witnessing or discovering) death or serious injury (23.7%). The next most common trauma types at the respondent level were muggings (14.5%), life-threatening automobile accidents (14.0%), and life-threatening illnesses (11.8%). ‘Private’ traumas were reported by 4.9% of respondents. When considered in terms of broader categories, the most common traumas at the respondent level were those that either occurred to a loved one or were witnessed (35.7% of respondents), those involving accidents (34.3%), and unexpected death of a loved one (31.4%) followed by physical violence (22.9%), intimate partner sexual violence (14.0%), war-related traumas (13.1%), and ‘other’ traumas (8.4%).

**Table 2.** Prevalence and distribution of lifetime traumas in the WMH Surveys (*n* = 68,894).

	Person-level		Mean exposures		Number of exposures		Each trauma type as a	
	lifetime prevalence^a^		given any^b^		per 100 people^c^		proportion of all traumas^d^	
	%	*(SE)*	Est	*(SE)*	%	*(SE)*	%	*(SE)*
I. War related trauma								
Combat experience	3.1	(0.1)	-	-	3.1	(0.1)	1.0	(0.0)
Purposely injured/killed someone	0.9	(0.1)	2.5	(0.1)	2.1	(0.1)	0.7	(0.0)
Saw atrocities	3.7	(0.1)	2.1	(0.0)	7.6	(0.2)	2.4	(0.1)
Relief worker or peacekeeper	1.0	(0.1)	-	-	1.0	(0.1)	0.3	(0.0)
Civilian in war zone	4.4	(0.1)	-	-	4.4	(0.1)	1.4	(0.0)
Civilian in region of terror	3.4	(0.1)	-	-	3.4	(0.1)	1.1	(0.0)
Refugee	2.2	(0.1)	-	-	2.2	(0.1)	0.7	(0.0)
Any	13.1	(0.2)	1.8	(0.0)	23.9	(0.5)	7.4	(0.1)
II. Physical violence								
Physically abused in childhood	7.9	(0.2)	-	-	7.9	(0.2)	2.5	(0.0)
Physically assaulted	5.9	(0.1)	2.0	(0.0)	11.5	(0.3)	3.6	(0.1)
Mugged	14.5	(0.2)	1.6	(0.0)	23.7	(0.4)	7.4	(0.1)
Kidnapped	1.1	(0.1)	1.1	(0.0)	1.2	(0.1)	0.4	(0.0)
Any	22.9	(0.3)	1.9	(0.0)	44.3	(0.6)	13.8	(0.2)
III. Intimate partner or sexual violence								
Physically abused by romantic partner	4.5	(0.1)	-	-	4.5	(0.1)	1.4	(0.0)
Raped	3.2	(0.1)	1.8	(0.0)	5.8	(0.2)	1.8	(0.1)
Sexually assaulted (other than raped)	5.8	(0.1)	2.0	(0.0)	11.7	(0.3)	3.6	(0.1)
Stalked	5.3	(0.1)	1.8	(0.0)	9.5	(0.2)	3.0	(0.1)
Any	14.0	(0.2)	2.3	(0.0)	31.5	(0.6)	9.8	(0.2)
IV. Accident								
Automobile accident	14.0	(0.2)	1.4	(0.0)	19.6	(0.3)	6.1	(0.1)
Other life-threatening accident	6.2	(0.1)	1.5	(0.0)	9.4	(0.2)	2.9	(0.1)
Natural disaster	7.4	(0.2)	1.8	(0.0)	13.1	(0.5)	4.1	(0.1)
Toxic chemical exposure	4.2	(0.1)	2.7	(0.1)	11.5	(0.4)	3.6	(0.1)
Other man-made disaster	4.0	(0.1)	1.7	(0.0)	6.9	(0.2)	2.1	(0.1)
Accidentally injured/killed someone	1.4	(0.1)	1.6	(0.1)	2.2	(0.1)	0.7	(0.0)
Life-threatening illness	11.8	(0.2)	1.4	(0.0)	16.5	(0.3)	5.1	(0.1)
Any	34.3	(0.3)	2.3	(0.0)	79.2	(1.1)	24.6	(0.2)
V. Unexpected death of a loved one								
Any	31.4	(0.3)	1.7	(0.0)	53.2	(0.7)	16.5	(0.2)
VI. Other traumas of loved ones or witnessed								
Child with serious illness	7.9	(0.1)	1.3	(0.0)	10.1	(0.2)	3.1	(0.1)
Other traumas to loved ones	5.6	(0.1)	1.5	(0.0)	8.5	(0.3)	2.7	(0.1)
Witnessed parenteral violence	7.9	(0.2)	-	-	7.9	(0.2)	2.5	(0.0)
Witnessed injury, death, dead body	23.7	(0.3)	2.3	(0.0)	53.9	(0.9)	16.8	(0.2)
Any	35.7	(0.3)	2.2	(0.0)	80.4	(1.0)	25.0	(0.2)
VII. Other traumas								
‘Other’ trauma	4.2	(0.1)	-	-	4.2	(0.1)	1.3	(0.0)
‘Private’ trauma	4.9	(0.1)	-	-	4.9	(0.1)	1.5	(0.0)
Any	8.4	(0.2)	1.1	(0.0)	9.1	(0.2)	2.8	(0.1)
VIII. Any	70.4	(0.3)	4.6	(0.0)	321.5	(2.9)	100.0	(0.0)

^a^The percent of all respondents who reported ever in their lifetime experiencing the trauma type indicated in the row heading. For example, 3.1% of respondents across surveys reported a history of combat experience.

^b^The mean number of lifetime occurrences of the trauma type indicated in the row heading among those who reported ever experiencing that trauma type. - entries indicate that we did not assess number of occurrences for the trauma type. For example, the respondents who reported ever in their life seriously injuring or killing someone on purpose reported a mean of 2.5 such occurrences.

^c^The number of lifetime occurrences of the trauma type indicated in the row heading per 100 respondents, which equals the product of the two earlier row entries. For example, the 2.5 lifetime occurrences of seriously injuring or killing someone on purpose reported by 0.9% of respondents results in 2.1 (0.9 × 2.5) lifetime occurrences of such a trauma for every 100 respondents in the sample.

^d^The ratio of the entry in the cell of the previous column to the 321.5 total lifetime traumas for every 100 respondents. For example, the 3.1 instances of combat experience represent approximately 1.0% of the 321.5 total.

The above results do not take into consideration the fact that mean number of exposures varies significantly across trauma types for the 20 traumas that were assessed for frequency (χ^2^_19_ = 11,729.9, *p* < .001). (The other trauma types were not assessed for frequency because they represented ongoing situations rather than discrete events.) When we multiply the proportion of respondents with any lifetime exposure to a given trauma type by mean number of exposures among those with any, we find a mean number of exposures to any trauma among people with any of 4.6. This translates into 3.2 lifetime trauma exposures per capita (i.e. .704 with any exposure × 4.6). The distribution of number of trauma occurrences among respondents with any was 25.8% one, 18.0% two, 12.9% three, 9.6% four, 7.0% five, 5.4% six, 4.2% seven, 3.2% eight, 2.4% nine, 2.2% 10, 4.7% 11–14, and 4.5% 15 or more. The most common traumas are those that happen to other people, either unexpected death of a loved one (16.5% of all traumas) or other traumas that happened to a loved one or that the respondent witnessed (25.0%), collectively accounting for over 40% of all trauma exposures. Another 24.6% are accidents and another roughly one-quarter involve either intimate partner sexual violence (9.8%) or physical violence (13.8%). The remaining categories of war-related (7.4%) and ‘other’ (2.8%) traumas are much less common.

### 3.2. Socio-demographic predictors of trauma exposure

Socio-demographic predictors of trauma exposure in the WMH data have been reported previously (Benjet et al., 2016). Using survival analysis, these analyses showed that women are much more likely than men to be exposed to intimate partner sexual violence (OR 2.3), roughly equal to men in odds of unexpected death of a loved one (OR 1.1), and significantly less likely than men to experience any of the other specific trauma types considered in our analysis (OR 0.4–0.8). Currently married respondents have significantly reduced odds of the vast majority of trauma types (OR 0.5–0.9) compared to the never married, while low education is associated with somewhat elevated risk of some (e.g. violence, accidents, natural disasters) but not all (e.g. unexpected death of a loved one) types of trauma.

**Figure 1.**
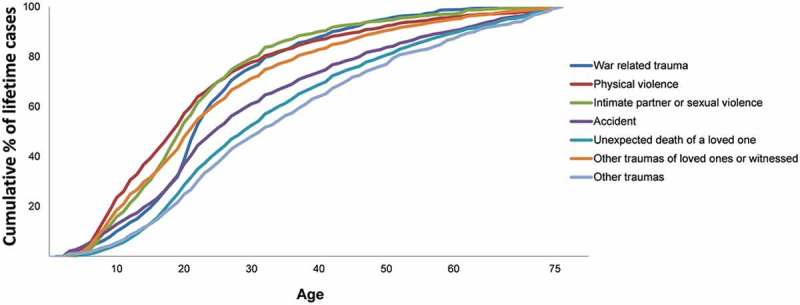
Age-of-onset distributions of trauma exposure in the WMH Surveys.

Perhaps the most interesting socio-demographic correlate of trauma exposure is age. Age-of-occurrence curves in Figure 1 show that traumas associated with interpersonal violence have earliest median age-of-occurrence (age 17) followed by intimate partner sexual violence (age 18), war-related traumas (age 20), and traumas that happened to other people (age 20). Accidents, unexpected death of loved ones, and other traumas have later median ages-of-occurrence (ages 24–31).

### 3.3. Differential risk of PTSD depending on trauma type

As detailed in another recent WMH report (Liu et al., 2017), conditional risk of DSM-IV/CIDI PTSD after trauma exposure is 4.0%, but varies significantly by trauma type. (Table 3) The highest conditional risk is associated with being raped (19.0%), physical abuse by a romantic partner (11.7%), being kidnapped (11.0%), and being sexually assaulted other than rape (10.5%). In terms of broader categories, the traumas associated with the highest PTSD risk are those involving intimate partner sexual violence (11.4%) and other traumas (9.2%), with aggregate conditional risk much lower in the other broad trauma categories (2.0–5.4%).

**Table 3.** Conditional risk of DSM-IV/CIDI PTSD by trauma category in the WMH Surveys.

	PTSD risk given		Number of PTSD episodes		Proportion of all PTSD episodes		
	trauma exposure^a^		per 100 people^b^		for each trauma type^c^		
	(%)	(*SE*)	Est	(*SE*)	%	(*SE*)	(*n*)
I. War related trauma							
Combat experience	3.6	(0.8)	0.1	(0.0)	0.9	(0.2)	(535)
Purposely injured/killed someone	4.0	(3.1)	0.1	(0.1)	0.7	(0.5)	(102)
Saw atrocities	5.4	(4.1)	0.4	(0.3)	3.2	(2.2)	(533)
Relief worker or peacekeeper	0.8	(0.7)	0.0	(0.0)	0.1	(0.1)	(139)
Civilian in war zone	1.3	(0.5)	0.1	(0.0)	0.5	(0.2)	(1050)
Civilian in region of terror	1.6	(0.6)	0.1	(0.0)	0.4	(0.1)	(634)
Refugee	4.5	(2.0)	0.1	(0.0)	0.8	(0.3)	(406)
Any	3.5	(1.4)	0.8	(0.3)	6.4	(2.3)	(3399)
II. Physical violence							
Physically abused in childhood	5.0	(1.0)	0.4	(0.1)	3.1	(0.6)	(2082)
Physically assaulted	2.5	(0.6)	0.3	(0.1)	2.2	(0.6)	(1201)
Mugged	1.8	(0.4)	0.4	(0.1)	3.4	(0.7)	(3277)
Kidnapped	11.0	(3.0)	0.1	(0.0)	1.0	(0.3)	(216)
Any	2.8	(0.4)	1.3	(0.2)	9.7	(1.2)	(6776)
III. Intimate partner or sexual violence							
Physically abused by romantic partner	11.7	(1.3)	0.5	(0.1)	4.1	(0.5)	(1675)
Raped	19.0	(2.2)	1.1	(0.1)	8.6	(1.0)	(1246)
Sexually assaulted (other than raped)	10.5	(1.5)	1.2	(0.2)	9.5	(1.3)	(1574)
Stalked	7.6	(2.0)	0.7	(0.2)	5.6	(1.4)	(1160)
Any	11.4	(1.0)	3.6	(0.3)	27.8	(2.0)	(5655)
IV. Accident							
Automobile accident	2.6	(0.4)	0.5	(0.1)	4.0	(0.7)	(3428)
Other life-threatening accident	4.9	(2.4)	0.5	(0.2)	3.5	(1.6)	(1205)
Natural disaster	0.3	(0.1)	0.0	(0.0)	0.3	(0.1)	(1669)
Toxic chemical exposure	0.1	(0.0)	0.0	(0.0)	0.1	(0.0)	(622)
Other man-made disaster	2.9	(1.3)	0.2	(0.1)	1.5	(0.7)	(726)
Accidentally injured/killed someone	2.8	(1.0)	0.1	(0.0)	0.5	(0.1)	(251)
Life-threatening illness	2.0	(0.3)	0.3	(0.1)	2.5	(0.4)	(3249)
Any	2.0	(0.3)	1.6	(0.3)	12.4	(1.9)	(11,150)
V. Unexpected death of loved one							
Any	5.4	(0.5)	2.9	(0.3)	22.2	(1.8)	(10,714)
VI. Other traumas of loved ones or witnessed							
Child with serious illness	4.8	(0.6)	0.5	(0.1)	3.8	(0.5)	(2452)
Other traumas to loved ones	5.1	(1.3)	0.4	(0.1)	3.4	(0.8)	(1173)
Witnessed parenteral violence	3.8	(0.7)	0.3	(0.1)	2.4	(0.4)	(2000)
Witnessed injury, death, dead body	1.3	(0.3)	0.7	(0.1)	5.5	(1.0)	(5114)
Any	2.4	(0.2)	1.9	(0.2)	15.0	(1.4)	(10,739)
VII. Other traumas							
‘Other’ trauma	9.1	(1.0)	0.4	(0.0)	3.0	(0.3)	(1260)
‘Private’ trauma	9.2	(1.1)	0.5	(0.1)	3.5	(0.4)	(1503)
Any	9.2	(0.7)	0.8	(0.1)	6.5	(0.5)	(2763)
VIII. Any	4.0	(0.2)	12.9	(0.7)	100	(0.0)	(51,196)

^a^The conditional risk of PTSD associated with the trauma type indicated in the row heading. For example, 3.6% of the combat experiences resulted in DSM-IV/CIDI PTSD.

^b^The mean number of lifetime episodes of PTSD associated with the trauma type indicated in the row heading per 100 respondents. For example, the 3.5% of lifetime war-related traumas that led to PTSD reported in the first column of Table 3, when multiplied by the 23.9 lifetime occurrences of such traumas per 100 respondents reported in the third column of Table 2, translates into 0.8 lifetime episodes of PTSD due to this category of traumas per 100 respondents.

^c^The ratio of the entry in the cell of the previous column to the total of 12.9 lifetime episodes of PTSD per 100 respondents. For example, the 0.8 cases of PTSD associated with war-related traumas represents 6.4% of the 12.9 total.

Prevalence of trauma exposure and conditional risk of PTSD both need to be considered in evaluating the trauma-specific population burden of PTSD. Given that 3.2 trauma exposures occur per capita in the population, the 4.0% aggregate conditional risk of PTSD translates into 12.9 lifetime episodes of PTSD per 100 people in the population. The trauma type associated with by far the highest number of these PTSD cases is unexpected death of a loved one (2.9 episodes of PTSD/100 population; 22.2% of all lifetime episodes of PTSD), with rape (1.1 episodes of PTSD/100 population; 8.6% of all lifetime episodes) and sexual assault other than rape (1.2 episodes of PTSD/100 population; 9.5% of all lifetime episodes) together accounting for another 18.1% of lifetime episodes. Unexpected death is a very common type of trauma (53.2 lifetime occurrences/100 population; 16.5% of all lifetime traumas) with a high-average conditional risk of PTSD (5.4%), whereas rape and other sexual assault are less common (5.8–11.7 lifetime occurrences/100 population; 1.8–3.6% of all lifetime traumas) with much higher conditional risks of PTSD (19.0–10.5%).

Four of the six trauma types associated with highest population proportions of lifetime PTSD episodes are in the category of intimate partner sexual violence. These include 4.1% of all lifetime PTSD episodes associated with physical abuse by a romantic partner, 8.6% with rape, 9.5% with other sexual assault, and 5.6% with being stalked, for a total of 27.8% of all lifetime episodes of PTSD. Intimate partner sexual traumas account for 9.8% of all lifetime trauma exposures and are associated with comparatively high conditional risk of PTSD. The only other trauma types accounting for as many cases of PTSD are the two most commonly-occurring traumas considered here: unexpected death of a loved one (22.2% of all cases of PTSD) which, as noted above, is the second most common trauma (16.5% of all traumas) associated with high-average conditional risk of PTSD, and direct exposure to death or serious injury (5.5% of all cases of PTSD), which is the most common trauma (16.8% of all traumas) and is associated with a comparatively low risk of PTSD (1.3%).

### 3.4. Socio-demographic predictors of PTSD conditional on trauma exposure

Controlling for trauma type, conditional PTSD risk is significantly associated with age, with risk highest during childhood–adolescence and ages 65+. Consistent with much previous research (reviewed by Olff, Langeland, Draijer, & Gersons, 2007; Tolin & Foa, 2006), women are significantly more likely to develop PTSD than are men exposed to the same traumas. We also looked at socio-economic status and marital status but found that they are not significant predictors of PTSD after controlling trauma type and respondent age–sex.

### 3.5. Associations of prior trauma exposure with subsequent PTSD

The literature suggests that people with a history of prior trauma exposure are more likely than others to develop PTSD after exposure to subsequent traumas (Breslau, Peterson, & Shultz, 2008; Caramanica et al., 2015). Consistent with this evidence, a previous WMH report found that the vast majority of prior trauma types are significantly and positively associated with subsequent trauma exposure (Benjet et al., 2016). The strongest of these associations (OR = 2.0–2.5) are for one type of physical violence (e.g. physical abuse in childhood) predicting other types of subsequent physical violence (e.g. being mugged) and intimate partner sexual violence.

As detailed in a recent WMH report (Liu et al., 2017), the WMH random trauma analysis replicated earlier studies in showing that history of prior trauma exposure predicts increased vulnerability to PTSD after subsequent traumas, but also went beyond previous studies in several important ways. First, this association was found to be limited to prior traumas involving physical or sexual violence (OR = 1.3–2.5). Second, the vulnerability to future PTSD associated with these prior traumas was found to be ‘generalized’ in the sense that it existed across the full range of random trauma types considered in the analysis. Third, there was evidence for two more specific types of vulnerability associated with prior lifetime exposure to the same trauma types as in the random traumas. One involved history of traumas involving physical violence, which was associated with significantly elevated odds of PTSD after subsequent re-exposure to the same trauma types (OR 3.2). This means that it is recurrent physical violence that is most strongly associated with high PTSD risk. The other involved history of traumas involving participation in sectarian violence (e.g. combat experience, purposefully injured or killed someone), which was associated with significantly reduced odds of PTSD after subsequent re-exposure to the same trauma types (OR 0.3). The last of these results might seem counter-intuitive given that military personnel and first responders working in situations of high trauma exposure are known to be at elevated risk of PTSD (Gates et al., 2012; Wilson, 2015). However, it is important to recognize that the result refers to PTSD after *re-exposure*, which is quite a different thing than PTSD after initial exposure. As reviewed below in the discussion section, this finding of prior experience helping to protect against the effects of sectarian violence is consistent with previous literature.

### 3.6. Persistence of PTSD symptoms

**Figure 2.**
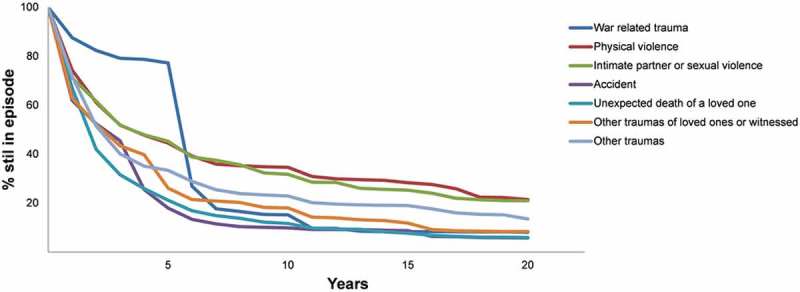
Speed of recovery of DSM-IV/CIDI PTSD by trauma category in the WMH Surveys.^1^ ^1^‘Recovery’ was defined as length of time until all symptoms remitted.

The results reported up to now focused on lifetime prevalence. However, population burden is more directly a function of point prevalence. And point prevalence is a joint function of lifetime prevalence and persistence. A recent comprehensive review of the literature on PTSD remission concluded that roughly half of PTSD cases remit within six months and that probability of remission does not vary dramatically across trauma types (Morina, Wicherts, Lobbrecht, & Priebe, 2014). We were able to investigate this issue in the WMH data by asking respondents with a history of PTSD associated with randomly-selected traumas to report on duration of symptoms and whether they still had symptoms at the time of interview. Mean duration of PTSD symptoms (Table 4) averages approximately six years (72.3 months) across all traumas but varies greatly depending on trauma type from a high of over 13 years for traumas involving combat experience in war to a low of about one year for traumas involving exposure to a natural disaster. It is noteworthy that WMH respondents were asked how long they continued to have *any* symptoms, so these duration estimates are for symptoms rather than for meeting full PTSD criteria. Speed-of-recovery curves (Figure 2) show that means in Table 4 are influenced by long right tails, with 25–40% of cases of PTSD recovering within one year, many of them within six months, the major exception being a much lower rate of rapid recovery among people with war-related PTSD. A smaller proportion of cases persists for many years. The longest median duration is five years for PTSD symptoms associated with war-related traumas followed by three years for traumas involving physical or intimate partner sexual violence. Median durations are one to two years, in comparison, for PTSD symptoms due to the other broad trauma categories.

**Table 4.** Mean duration and years in episode of DSM-IV/CIDI PTSD by trauma type in the WMH Surveys.

	Mean PTSD episode duration		Number of years with		Proportion of all years with		
	(in months) by trauma type^a^		PTSD per 100 people^b^		PTSD for each trauma type^c^		
	Est	(*SE*)	Est	(*SE*)	%	(*SE*)	(*n*)
I. War related trauma							
Combat experience	161.7	(23.3)	1.5	(0.3)	1.9	(0.5)	(54)
Purposely injured/killed someone	79.3	(8.9)	0.6	(0.4)	0.7	(0.5)	(7)
Saw atrocities	78.3	(22.1)	2.7	(1.6)	3.5	(2.0)	(29)
Relief worker or peacekeeper	95.3	(45.8)	0.1	(0.0)	0.1	(0.1)	(2)
Civilian in war zone	62.9	(26.7)	0.3	(0.1)	0.4	(0.2)	(29)
Civilian in region of terror	38.5	(15.6)	0.2	(0.0)	0.2	(0.0)	(20)
Refugee	44.7	(20.2)	0.4	(0.1)	0.5	(0.2)	(20)
Any	82.0	(11.8)	5.7	(1.8)	7.3	(2.2)	(161)
II. Physical violence							
Physically abused in childhood	138.6	(25.7)	4.6	(0.7)	5.9	(1.0)	(174)
Physically assaulted	22.7	(4.6)	0.5	(0.1)	0.7	(0.2)	(62)
Mugged	115.0	(47.5)	4.2	(2.3)	5.4	(2.8)	(119)
Kidnapped	115.9	(38.1)	1.3	(0.5)	1.6	(0.7)	(40)
Any	101.4	(18.6)	10.6	(2.6)	13.6	(3.0)	(395)
III. Intimate partner or sexual violence							
Physically abused by romantic partner	82.7	(9.0)	3.6	(0.5)	4.7	(0.7)	(318)
Raped	110.3	(14.1)	10.2	(1.4)	13.1	(1.9)	(443)
Sexually assaulted (other than raped)	114.2	(18.5)	11.7	(2.1)	15.1	(2.7)	(280)
Stalked	127.1	(84.2)	7.6	(6.0)	9.8	(6.5)	(103)
Any	110.9	(18.0)	33.2	(6.5)	42.7	(4.9)	(1144)
IV. Accident							
Automobile accident	52.5	(15.0)	2.2	(0.7)	2.9	(0.9)	(170)
Other life-threatening accident	28.6	(6.4)	1.1	(0.6)	1.4	(0.8)	(41)
Natural disaster	12.9	(4.3)	0.0	(0.0)	0.0	(0.0)	(22)
Toxic chemical exposure	40.4	(22.2)	0.0	(0.0)	0.1	(0.0)	(9)
Other man-made disaster	41.3	(8.4)	0.7	(0.3)	0.9	(0.4)	(31)
Accidentally injured/killed someone	54.5	(27.7)	0.3	(0.1)	0.4	(0.1)	(24)
Life-threatening illness	41.3	(9.0)	1.1	(0.3)	1.4	(0.3)	(152)
Any	41.2	(5.7)	5.5	(1.0)	7.1	(1.4)	(449)
V. Unexpected death of a loved one							
Any	37.7	(3.9)	9.0	(0.9)	11.6	(1.5)	(1158)
VI. Other traumas of loved ones or witnessed							
Child with serious illness	44.7	(8.7)	1.8	(0.4)	2.3	(0.5)	(225)
Other traumas to loved ones	45.7	(12.8)	1.7	(0.5)	2.1	(0.6)	(101)
Witnessed parenteral violence	107.1	(15.0)	2.7	(0.5)	3.5	(0.7)	(135)
Witnessed injury, death, dead body	45.4	(9.8)	2.7	(0.8)	3.5	(1.0)	(140)
Any	55.0	(5.1)	8.9	(1.1)	11.4	(1.6)	(601)
VII. Other traumas							
‘Other’ trauma	62.1	(12.1)	2.0	(0.4)	2.5	(0.6)	(201)
‘Private’ trauma	79.5	(13.1)	3.0	(0.4)	3.9	(0.6)	(248)
Any	71.5	(6.8)	5.0	(0.6)	6.4	(0.9)	(449)
VIII. Any	72.3	(6.0)	77.7	(7.5)	100.0	(0.0)	(4357)

^a^The mean duration (in months) of PTSD episodes associated with the trauma type indicated in the row heading. Recovery was defined as the number of months until the respondent stopped having any symptoms. For example, respondents with a history of PTSD due to combat experience reported that symptoms continued for a mean of 161.7 months (13.5 years).

^b^The number of lifetime episodes of PTSD due to the trauma type indicated in the row heading per 100 respondents from the third column in Table 3 multiplied by the mean duration (in years) from the first column of Table 4. For example, the 0.7 lifetime episodes of PTSD due to war-related traumas per 100 respondents multiplied by the mean 6.8 years per episode results in 5.7 (0.8 × 6.8) years of PTSD due to this category of traumas per 100 respondents.

^c^The ratio of the entry in the cell of the previous column to the total 77.7 years of PTSD due to any trauma for every 100 respondents. For example, the 5.7 years of war-related PTSD represent 7.3% of the 77.7 total.

We also looked for socio-demographic predictors of PTSD symptom duration. None were significant in the total sample of cases. However, we lacked the statistical power to investigate the possibility that these predictors vary depending on trauma type. Such variation has been documented in focused studies. For example, a large prospective study of victims of Hurricane Katrina found that socio-economic status was a significant predictor of speed of PTSD recovery after that natural disaster (McLaughlin et al., 2011). Previous research on the predictors of recovery in trauma-specific samples have focused largely on trauma characteristics and prior psychopathology (Atwoli et al., 2017; Bromet et al., 2017; Stein et al., 2016), neither of which was considered in the aggregate WMH analyses due to the small numbers of cases associated with each trauma type.

Our most direct estimate of the population burden of PTSD associated with each trauma type is the number of years of PTSD at the population level associated with that trauma type. An estimate of the latter can be obtained by multiplying number of lifetime PTSD episodes/100 population by mean duration. When this is done and the trauma-specific products are summed across all trauma types we estimate that there are 77.7 lifetime person-years of PTSD in the population per 100 respondents. The four trauma types with the highest proportions of these person-years are rape (13.1%; 10.2 person-years per 100 respondents), other sexual assault (15.1%; 11.7 person-years per 100 respondents), being stalked (9.8%; 7.6 person-years per 100 respondents), and unexpected death of a loved one (11.6%; 9.0 person-years per 100 respondents). The broad category of intimate partner sexual violence accounts for nearly 42.7% of all person-years with PTSD in the population (33.2 person-years per 100 respondents).

## 4. Discussion

The WMH results are limited in a number of ways. For one, lifetime prevalence estimates of trauma exposure are likely to be conservative due to recall error (Belli, 2014). In addition, some traumas are likely to be systematically under-reported because they are embarrassing or otherwise culturally sensitive (Schaeffer, 2000). Both types of problems can be reduced, although not entirely overcome, with data collection enhancements. Recall failure can be reduced by using memory priming strategies and event history calendars to focus memory search (Drasch & Matthes, 2013). Conscious nondisclosure can be reduced by increasing anonymity; for example, by having respondents privately record sensitive information in a self-report booklet that is sealed before returning it to the interviewer or via private computerized self-administration (Gnambs & Kaspar, 2015). There is some concern that complete anonymity can reduce motivation to report accurately (Lelkes, Krosnick, Marx, Judd, & Park, 2012). These strategies were not used in the WMH Surveys, so we have to consider the WMH trauma exposure prevalence estimates as lower-bound estimates.

Another limitation of the WMH results involves the diagnoses of lifetime PTSD. These diagnoses are limited by being based on retrospective reports obtained in a cross-sectional survey using a fully-structured lay-administered diagnostic interview rather than a semi-structured clinician-administered diagnostic interview. The WMH clinical reappraisal study shows that PTSD prevalence is under-estimated in the CIDI compared to blinded semi-structured clinical interviews but that the vast majority of CIDI cases are confirmed in these clinical reappraisal interviews (Haro et al., 2006), suggesting that the WMH prevalence estimates are conservative. WMH results regarding PTSD persistence, in comparison, are anti-conservative because they assess persistence of any symptom rather than of the full PTSD syndrome. Results regarding predictors, finally, are limited by excluding prior psychopathology, which is known to be the strongest predictor of PTSD onset given trauma type (DiGangi et al., 2013; Sayed et al., 2015), and trauma characteristics-sequelae, which are known to be strong predictors of persistence (Morina et al., 2014).

Within the context of these limitations, our finding that 70.4% of respondents were exposed to one or more traumas at some time in their life is broadly consistent with previous research reviewed elsewhere (Benjet et al., 2016) documenting that the majority of people in the general population have experienced traumas. However, WMH went beyond previous studies in assessing frequency of exposure, documenting that trauma exposure is even more common than previously known, with a per capita mean of 2.0 trauma types and 4.6 trauma exposures. These estimates are conservative as they are based on calculations in which some ongoing traumas, such as physical abuse at the hands of a caregiver during childhood, are counted as only ‘one occurrence’ even though they often persisted over many years.

WMH results regarding the most common types of trauma are consistent with previous research in finding that unexpected death of a loved one and motor vehicle accidents are the two most common types of trauma in the general population (reviewed by Benjet et al., 2016). We went beyond these previous results to show that traumas occurring to other people account for over 40% of all reported qualifying (for a diagnosis of PTSD) traumas (16.5% involving unexpected death of a loved one and an additional 25.0% other traumas that either occurred to a loved one or were witnessed), that accidents are the most common type of trauma occurring to people directly (24.6%), and that traumas involving intimate partner sexual violence (9.8%) and physical violence (13.8%) account for the bulk of other traumas. It is noteworthy that the objective occurrence of traumas to loved ones is clearly under-reported by WMH respondents in that we would expect each loved one of each respondent to have as many traumas as the respondent himself or herself, but this is not the case in respondent reports, implying that respondent reports about traumas occurring to loved ones are limited to the people and traumas most psychologically salient to respondents.

We also found that trauma exposure is not distributed randomly in the population. Our results are consistent in this regard with a previous study that reviewed the literature on basic socio-demographic correlates of trauma exposure (Hatch & Dohrenwend, 2007) in finding that women are significantly more likely than men to experience intimate partner sexual violence and men more likely than women to experience physical violence and accidents. We found that traumas involving violence and accidents are more likely to occur in adolescence and early adulthood that other parts of the life course. We also found that being married is the most consistent socio-demographic factor associated with reduced risk of many types of trauma exposure, while traumas involving violence and accidents (including natural disasters) are inversely associated with socio-economic status. And we found that trauma exposures are correlated over time, with people exposed to earlier traumas at significantly increased risk of subsequent traumas. The latter pattern presumably reflects individual differences in predispositions, coping resources, life circumstances, and lifestyles that influence risk of trauma exposure. The WMH data were too coarse to search for modifiable risk factors that might be targeted to prevent future trauma exposure, but the strong inter-temporal patterning of exposure suggests that such an investigation might make sense. Preventive interventions with this focus already exist for recurrences of drunk driving (Miller, Curtis, Sønderlund, Day, & Droste, 2015), intimate partner violence (Ramsay et al., 2009), and sexual violence (Marques, Wiederanders, Day, Nelson, & Ommeren, 2005), but our results raise the possibility of also developing risk models to target broader types of secondary preventive interventions.

Our estimates of conditional PTSD risk among people exposed to traumas are for the most part lower than in previous studies due to our focus on representative samples of traumas in comparison to the worst traumas examined in most other community epidemiological studies and in samples that over-represent help-seekers focused on particular trauma types (e.g. Campbell, Dworkin, & Cabral, 2009; Goldmann & Galea, 2014).

Our finding that conditional PTSD risk is elevated after traumas involving violence is broadly consistent with previous research (see reviews in Atwoli et al., 2015; Ozer, Best, Lipsey, & Weiss, 2003). We also found that prior exposure to some traumas involving violence was associated with *generalized vulnerability* to subsequent PTSD. Although ongoing research is investigating pathways leading to such generalized vulnerability (Daskalakis, Bagot, Parker, Vinkers, & de Kloet, 2013; Levy-Gigi, Richter-Levin, Okon-Singer, Kéri, & Bonanno, 2016; Rutter, 2012), we know of no work on differential effects of trauma types in this regard. However, suggestive related evidence exists on differences in associations of childhood adversities with adult mental disorders across different childhood adversity types (Kessler et al., 2010; Pirkola et al., 2005) and profiles (McLafferty et al., 2015; Putnam, Harris, & Putnam, 2013).

Our finding that prior same-type physical violence victimizations predict elevated PTSD risk after re-victimization means that these types of victimization are especially impactful when they are recurrent. That being the case, a question can be raised about our failure to find a similar pattern for intimate partner sexual violence, as the latter seems to contradict studies showing that sexual assault *re-victimization* is associated with poor mental health (Classen, Palesh, & Aggarwal, 2005; Das & Otis, 2016; Miner, Flitter, & Robinson, 2006). However, these studies focused largely on victims of childhood sexual assault who were versus were not re-victimized as adults, whereas our analysis compares adult sexual assault victims who were versus were not previously victimized.

Our finding that prior same-type participation in sectarian violence is associated with low PTSD risk after subsequent re-exposure to the same trauma is consistent with research showing low PTSD prevalence among policemen (Levy-Gigi et al., 2016) and other first responders (Levy-Gigi & Richter-Levin, 2014) and among Israeli settlers exposed to repeated bombings (Palgi, Gelkopf, & Berger, 2015; Somer et al., 2009). These results could be due either to selection and/or to prior exposures promoting resilience (Wilson et al., 2009). Both experimental animal studies (Liu, 2015) and observational human studies (Rutter, 2012) support the resilience possibility, although research showing that intervening psychopathology due to prior traumas mediates the association between trauma history and subsequent PTSD (Sayed et al., 2015) confirms that prior traumas are more likely to create vulnerability than resilience. Research on the ‘healthy warrior effect’ supports the selection possibility (Larson, Highfill-McRoy, & Booth-Kewley, 2008; Wilson et al., 2009). As a result, we suspect that both processes are at work (i.e. both selection and environmental causation), although we have no way to estimate their relative importance with the WMH data.

Our results regarding persistence are broadly consistent with previous studies in showing that a substantial minority of PTSD cases remits within months after onset. However, we found that median duration of symptoms was longer than the six months found in a recent review of the literature (Morina et al., 2014). This reflects upward bias in the WMH data due to the very narrow definition of remission used in WMH, which required the respondent no longer to have *any* PTSD symptoms, leading to artificially high estimates of duration. This means that duration of sub-threshold PTSD is considerably longer than generally appreciated. Given that sub-threshold PTSD has been shown to be associated with considerable distress, impairment, and comorbidity (McLaughlin et al., 2015), the latter possibility is worthy of future investigation in prospective studies.

## 5. Conclusions

The WMH data document clearly that trauma exposure is common throughout the world, that this exposure is unequally distributed in the population, and that PTSD risk differs substantially across trauma types due to traumas involving interpersonal violence (especially relationship–sexual violence) carrying the highest PTSD risk. There is also high population-level burden of PTSD associated with unexpected death of a loved one, a very common trauma type that is associated with low individual-level PTSD risk. Although a substantial minority of PTSD cases remits within months after onset, mean symptom duration is considerably longer than previously recognized.

The WMH Surveys were designed as needs assessment surveys to help governments gain insights into the population burden of mental disorders. Because of this, the most important implications of the results are for policy planners in recognizing that PTSD is a very commonly-occurring condition. Although we did not present any results about severity of illness, other WMH results document clearly that PTSD is a seriously impairing disorder (Kessler et al., 2009). PTSD causes substantial loss of human capital from a societal perspective both in the form of days out of role (Alonso et al., 2011) and in the form of decreased productivity on days in role (Ormel et al., 2008). Roughly half of people with PTSD in high income countries and about half that number in low or middle income countries seek some type of treatment (Koenen et al., 2017), but the type and duration of treatment seldom meet even minimal standards for treatment adequacy (Wang et al., 2007). These results suggest that outreach efforts are needed to increase the proportion of people with PTSD who obtain treatment and that treatment quality improvement efforts are needed for patients in treatment.

## Supplementary Material

Spanish abstract

## Highlights of article

- Lifetime trauma exposure is the norm in most countries.
- Interpersonal violence traumas carry highest PTSD risk.
- Lifetime population burden of PTSD is 77.7 person-years/100 respondents across surveys.
- Trauma types with highest PTSD burden include those involving intimate partner sexual violence (relatively uncommon traumas associated with high PTSD risk) and unexpected death of a loved one (a very common trauma associated with low PTSD risk).
- Although many cases remit within months, PTSD symptoms typically are quite persistent.

## Group Information

The WHO World Mental Health Survey collaborators are Sergio Aguilar-Gaxiola, MD, PhD, Ali Al-Hamzawi, MD, Mohammed Salih Al-Kaisy, MD, Jordi Alonso, MD, PhD, Laura Helena Andrade, MD, PhD, Corina Benjet, PhD, Guilherme Borges, ScD, Evelyn J. Bromet, PhD, Ronny Bruffaerts, PhD, Brendan Bunting, PhD, Jose Miguel Caldas de Almeida, MD, PhD, Graça Cardoso, MD, PhD, Somnath Chatterji, MD, Alfredo H. Cia, MD, Louisa Degenhardt, PhD, Koen Demyttenaere, MD, PhD, John Fayyad, MD, Silvia Florescu, MD, PhD, Giovanni de Girolamo, MD, Oye Gureje, MD, DSc, FRCPsych, Josep Maria Haro, MD, PhD, Yanling He, MD, Hristo Hinkov, MD, PhD, Chi-yi Hu, MD, PhD, Yueqin Huang, MD, MPH, PhD, Peter de Jonge, PhD, Aimee Nasser Karam, PhD, Elie G. Karam, MD, Norito Kawakami, MD, DMSc, Ronald C. Kessler, PhD, Andrzej Kiejna, MD, PhD, Viviane Kovess-Masfety, MD, PhD, Sing Lee, MB, BS, Jean-Pierre Lepine, MD, Daphna Levinson, PhD, John McGrath, MD, PhD, Maria Elena Medina-Mora, PhD, Jacek Moskalewicz, PhD, Fernando Navarro-Mateu, MD, PhD, Beth-Ellen Pennell, MA, Marina Piazza, MPH, ScD, Jose Posada-Villa, MD, Kate M. Scott, PhD, Tim Slade, PhD, Juan Carlos Stagnaro, MD, PhD, Dan J. Stein, FRCPC, PhD, Margreet ten Have, PhD, Yolanda Torres, MPH, Dra.HC, Maria Carmen Viana, MD, PhD, Harvey Whiteford, MBBS, PhD, David R. Williams, MPH, PhD, Bogdan Wojtyniak, ScD.
