# Supplementary material for: Trauma and PTSD in the WHO World Mental Health Surveys
Source: Eur J Psychotraumatol. 2017 Oct 27;8(sup5):1353383. doi: 10.1080/20008198.2017.1353383 (PMC5632781; doi:10.1080/20008198.2017.1353383)
Supplement: Spanish abstract [file ZEPT_A_1353383_SM3527.pdf]

Planteamiento: Aunque se cree que la aparición y la persistencia del TEPT a lo largo de la vida varían significativamente según el tipo de trauma, la mayoría de las encuestas epidemiológicas a gran escala son incapaces de evaluar esta posibilidad porque evalúan el TEPT solo para los traumas que los encuestados consideran “el peor”.

Objetivo: Revisar las investigaciones sobre las asociaciones de los tipos de trauma con el TEPT en las encuestas de la OMS sobre la salud mental en el mundo, una serie de encuestas epidemiológicas comunitarias que aportaron datos representativos sobre el TEPT específico del trauma.

Método: Las encuestas sobre la salud mental en el mundo en 24 países (n = 68.894) evaluaron 29 traumas vitalicios y evaluaron dos veces el TEPT por cada encuestado: una vez para el "peor" trauma y, por separado, para un trauma seleccionado al azar con un peso que ajustase las diferencias individuales en las exposiciones al trauma. Se evaluó el inicio y la persistencia del TEPT con la *Composite International Diagnostic Interview* (CIDI) de la OMS.

Resultados: El 70,4% de los encuestados en la muestra consolidada experimentaron traumas vitalicios, con una exposición promedio de 3,2 traumas per cápita. Se encontraron diferencias sustanciales entre traumas en cuanto al inicio del TEPT, pero menos en cuanto a la persistencia. Los traumas que implican violencia interpersonal tenían mayor riesgo. La carga del TEPT, que se determina multiplicando la prevalencia del trauma por el riesgo de TEPT específico de trauma y la persistencia, fue de 77,7 personas-años por cada 100 encuestados. Los tipos de trauma con las proporciones más altas de esta carga fueron violación (13,1%), otra agresión sexual (15,1%), acoso (9,8%) y muerte inesperada de un ser querido (11,6%). Los tres primeros de estos cuatro representan traumas relativamente infrecuentes con alto riesgo de TEPT y el último, un trauma muy frecuente con bajo riesgo de TEPT. La amplia categoría de violencia sexual asociada a la pareja representó casi el 42,7% de todos las personas-años con TEPT. Una historia previa de trauma predijo tanto la exposición futura al trauma como el riesgo de TEPT en el futuro.

Conclusiones: La exposición al trauma es común en todo el mundo, está distribuida de manera desigual y existen diferencias entre los tipos de trauma con respecto al riesgo de TEPT. Aunque una minoría sustancial de casos de TEPT remite a los pocos meses de su aparición, la duración media de los síntomas es considerablemente más larga de lo que se había reconocido previamente.
